# Determination of Motion Parameters of Selected Major Tectonic Plates Based on GNSS Station Positions and Velocities in the ITRF2014

**DOI:** 10.3390/s21165342

**Published:** 2021-08-07

**Authors:** Marcin Jagoda

**Affiliations:** Faculty of Civil Engineering, Environmental and Geodetic Sciences, Koszalin University of Technology, Śniadeckich 2, 75-453 Koszalin, Poland; marcin.jagoda@tu.koszalin.pl

**Keywords:** GNSS stations, tectonic plate motion parameters, ITRF

## Abstract

Current knowledge about tectonic plate movement is widely applied in numerous scientific fields; however, questions still remain to be answered. In this study, the focus is on the determination and analysis of the parameters that describe tectonic plate movement, i.e., the position (Φ and Λ) of the rotation pole and angular rotation speed (*ω*). The study was based on observational material, namely the positions and velocities of the GNSS stations in the International Terrestrial Reference Frame 2014 (ITRF2014), and based on these data, the motion parameters of five major tectonic plates were determined. All calculations were performed using software based on a least squares adjustment procedure that was developed by the author. The following results were obtained: for the African plate, Φ = 49.15 ± 0.10°, Λ = −80.82 ± 0.30°, and *ω* = 0.267 ± 0.001°/Ma; for the Australian plate, Φ = 32.94 ± 0.05°, Λ = 37.70 ± 0.12°, and *ω* = 0.624 ± 0.001°/Ma; for the South American plate, Φ = –19.03 ± 0.20°, Λ = −119.78 ± 0.39°, and *ω* = 0.117 ± 0.001°/Ma; for the Pacific plate, Φ = −62.45 ± 0.07°, Λ = 111.01 ± 0.14°, and *ω* = 0.667 ± 0.001°/Ma; and for the Antarctic plate, Φ = 61.54 ± 0.30°, Λ = −123.01 ± 0.49°, and *ω* = 0.241 ± 0.003°/Ma. Then, the results were compared with the geological plate motion model NNR-MORVEL56 and the geodetic model ITRF2014 PMM, with good agreement. In the study, a new approach is proposed for determining plate motion parameters, namely the sequential method. This method allows one to optimize the data by determining the minimum number of stations required for a stable solution and by identifying the stations that negatively affect the quality of the solution and increase the formal errors of the determined parameters. It was found that the stability of the solutions of the Φ, Λ, and *ω* parameters varied depending on the parameters and the individual tectonic plates.

## 1. Introduction

Since the beginning of the Earth, its surface has been undergoing dynamic changes, which are caused both by external forces as well as those that act inside the planet, and their effects are visible on the surface. One such dynamic change process is tectonic plate movements on the surface of the asthenosphere. Current knowledge of the motion of the plates has been applied in numerous areas of research such as environmental sciences. Processes that result from plate tectonics, such as seismic or volcanic activity, have a direct influence on the environment. An issue that has been recently discussed by McEvoy et al. [1] is the influence of the movement of the lithosphere on the safety of radioactive waste stored underground. In addition, in [2,3], the authors analysed the influence of plate tectonics on climate change. Another research area where knowledge of tectonic plate motion has been applied is geodynamics and geodesy, in particular, related to defining the Earth’s reference systems [4,5]. For years, a problem for studies focused on lithospheric deformations, has been the formulation of laws that govern these phenomena, in particular, explaining the driving mechanism of this motion, and thus the dynamics of the lithosphere. A pioneer of the idea of lithospheric motion, based on the adherence of the coastal lines of both Americas, Africa, and Europe, was A. Snider-Pellegrini [6]. However, only the arguments later provided by A. Wegener [7] contributed to the fact that the latter is considered to be the author of the foundation of the lithospheric motion theory. Currently, studies in the literature, for example, studies by [8,9], divide the lithosphere into seven major plates of various sizes (Eurasian, African, North American, South American, Australian, Pacific, and Antarctic), and several smaller ones, referred to as minor plates and microplates, which are parts of the major plates or complement them. Most of the plates are located along the western margin of the Pacific Ocean, as detailed in [10] and more detailed information about tectonic plate movement can be found, for example, in [11,12]. The 1980s was a period that witnessed dynamic development of space measurement techniques, including satellite laser ranging (SLR), doppler orbitography and radiopositioning integrated by satellite (DORIS), very long baseline interferometry (VLBI), and global navigation satellite systems (GNSS). These techniques have started to be used for precise determination of tectonic plate motion, due to the possibility of conducting observations at a global scale. Nowadays, the SLR, DORIS, VLBI, and GNSS techniques are the basis for studies on crustal movements [13], as they allow plate movements to be quantified at a level of submillimetres per year. Data from satellite systems are the basis for creating kinematic models of plate movements, referred to as plate kinematic and crustal deformation models, such as the series of models developed by H. Drewes [14,15,16,17,18,19,20,21]. Then, the obtained results have been compared with the geological models of plate movement that have been developed based on geophysical data. Examples of such models include: AMO-2 [22], NNR-NUVEL-1 [23], NNR-NUVEL-1A [24], PB2002 [25], and the newest one, NNR-MORVEL56 [26]. Conducting studies on a global scale requires a reference system that is uniform in geometrical and physical terms for the whole Earth. The International Terrestrial Reference System (ITRS) is such a reference system, and an International Terrestrial Reference Frame (ITRF) is a realization of the ITRS. To date, the International Earth Rotation and Reference System Service (IERS) [27] has realised more than ten solutions of the ITRF system, among which the currently valid one is the ITRF2014 [28]. The ITRF2014 was generated using the complete observation history of the four space techniques SLR, DORIS, VLBI, and GNSS. The corresponding international services, i.e., the International Laser Ranging Service [29], the International DORIS Service [30], the International VLBI Service [31], and the International GNSS Service [32], provided reprocessed time series of station positions and daily Earth orientation parameters (EOP). The International GNSS Service submitted time series comprising 7714 daily solutions, resulting from the second reprocessing campaign, covering the time period 1994.0–2015.1 [33]. It is worthwhile noting, here, that another realisation of the ITRF, namely the ITRF2020, should be available by the end of 2021. More details regarding specifications of the ITRF solutions can be found in [34,35] or IERS Technical Note 36 [36].

In order to meet the expectations of the users of various geodynamic, geodetic, and environmental applications, the realisations of the ITRF have been accompanied by the publication of a tectonic plate motion model, for example, APKIM2005 [19], ITRF2008 PMM [4], and ITRF2014 PMM [5]. These are kinematic models that have been created based on the position (coordinates) and velocities of the SLR, DORIS, VLBI, and GNSS stations in the given ITRF frame as one common solution (SLR + DORIS + VLBI + GNSS).

Considering the need for continuous analysis of tectonic plate movement due to the demands of contemporary geodynamics, geodesy, and the influence on the environment, this study was conducted to determine and analyse plate motion parameters based on the coordinates and velocities of SLR, DORIS, VLBI, and GNSS stations, separately, for each of these techniques. Such an approach offers the possibility to evaluate the contribution of each of these space techniques to the creation of a model of movement of specific tectonic plates, and to assess the accuracy, of each technique, for determining the plate motion parameters. In this study, a new approach is proposed for determining tectonic plate motion parameters, namely the sequential method, described in Section 2. It is successfully demonstrated that this method can be used to identify, and then to eliminate, from the calculations, the stations which, due to various reasons (e.g., being located on cracked, unstable areas of the given plate, areas of seismic activity, or on a microplate, so their movement is not consistent with the motion of the analysed plate), contribute to an increase in formal errors of the determined parameters and the effects on the determination results. The method also allows for data optimisation, i.e., specifying the minimum number of stations on the given tectonic plate for which the calculated motion parameters are stabilised (i.e., their changes do not exceed the value of formal errors). To date, studies have been carried out on the SLR [37,38], DORIS [38,39], and VLBI [38,40] techniques and, on the GNSS technique, for the Eurasian plate [41]. In this study, the subsequent stage of the conducted studies is presented. The aims of the study are: (i) to determine and analyse the motion parameters of the African, Australian, South American, Pacific, and Antarctic plates based on the coordinates and velocities of the GNSS stations in the ITRF2014; (ii) to identify the stations that increase the value of formal errors in the defined parameters and negatively affect the calculation results; and (iii) to estimate the stability of the solutions of the motion parameters of a given plate.

## 2. Materials and Methods

**Figure 1:**
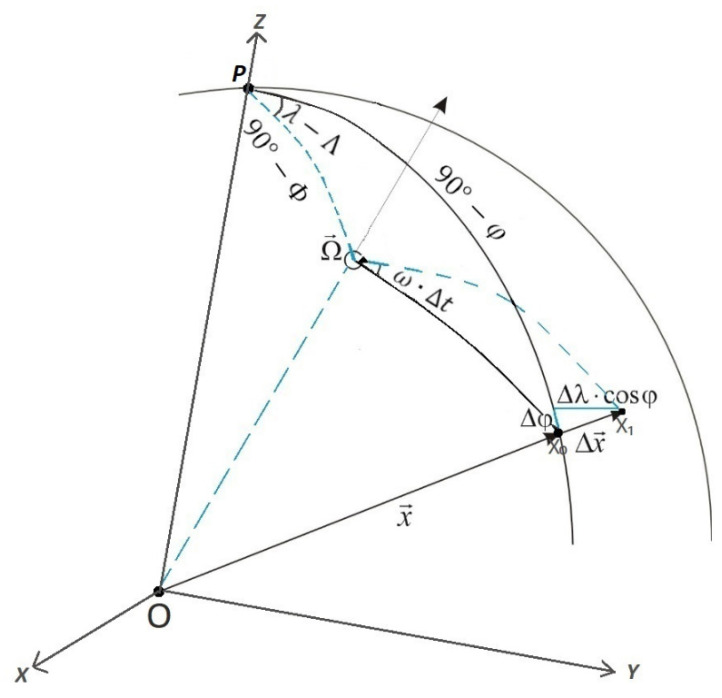
Relations between $\;\vec{\Omega }\;$ (Φ, Λ, and *ω*) and $\Delta \vec{x}\;$(Δ*ϕ* and Δ*λ*).

The data provided by SLR, DORIS, VLBI, and GNSS are the basis for the determination of the positions *ϕ* and *λ* of the observation stations of these techniques. The positions of the stations are subject to changes in time as a result of, among others, the movement of the tectonic plates on which they are located. According to the station positions *ϕ* and *λ* determined at time intervals Δ*t*, the movement of the station in time $\Delta \vec{x}$ can be calculated. Then, knowing the value of the displacement $\Delta \vec{x}$ (in terms of coordinate shifts Δ*ϕ* and Δ*λ*) of the station on a given plate, we can determine three parameters that describe the movement of this plate, i.e., the geographical latitude Φ and longitude Λ of the pole of rotation Ω and the angular rotation speed *ω* of the given plate or the specific elements of the pole of rotation *ω*_x_, *ω*_y_, and *ω*_z_ around the *X*, *Y*, and *Z* axes (Figure 1). The relations between these values are described in Equation (1) [42]. The geometric relations between the plate motion parameters and coordinate shifts are shown in Figure 1, where $X_{0}$ is the position of the station on the initial epoch $t_{0}$, $X_{1}$ is the position of the station on the epoch $t_{1}$ (after plate moving), and point *P* denotes the Earth pole. Equation (1):

(1) $$\begin{array}{c} \tan\Phi =\frac{\omega_{z}}{\sqrt{\omega_{x}^{2}+\omega_{y}^{2}}}\\ \tan\Lambda =\frac{\omega_{y}}{\omega_{x}}\\ \omega =\sqrt{\omega_{x}^{2}+\omega_{y}^{2}+\omega_{z}^{2}}\\ \omega_{x}=\omega \cos\Lambda \cos\Phi \\ \omega_{y}=\omega \sin\Lambda \cos\Phi \\ \omega_{z}=\omega \sin\Phi \end{array}$$

According to [17], the displacement of the observational station $\Delta \vec{x}=\left(\;\vec{\Omega }\;\times \vec{x}\right)\Delta t$ expressed as a function of the tectonic plate motion parameters Φ, Λ, *ω* (Δφ=f(Φ,Λ,ω), Δλ=f(Φ, Λ, ω)) is described by Equation (2):

(2) $$\begin{array}{c} \Delta \varphi =\omega \cdot \Delta t\cdot \cos\Phi \cdot \sin\left(\lambda -\Lambda \right)\\ \Delta \lambda =\omega \cdot \Delta t\cdot \left(\sin\Phi -\cos\left(\lambda -\Lambda \right)\cdot \tan\varphi \cdot \cos\Phi \right) \end{array}$$

Determining the motion parameters of a tectonic plate requires knowledge of the coordinates and velocities of at least two stations on that plate. This allows for the creation of two observational equations, i.e., Equation (3), and for the determination of three plate motion parameters Φ, Λ, and *ω*. In such a case (when the number of observational equations is higher than the number of the determined unknowns) it may be aligned with the use of the least-squares adjustment method. Assuming that more than two stations are located on a given plate allows one to evaluate the influence of the number and location of the stations on the determined motion parameters of the plate and the accuracy of their determination. A description of the least squares adjustment procedure was presented, among others, by McCarthy et al. [43], and its practical applications for the determination of plate motion parameters in [14], and in the earlier study by Jagoda et al. [40].

According to [14], the observational equations for the least squares adjustment procedure, which allow for the determination of plate motion parameters based on shifts in the station coordinates are expressed in Equation (3):

(3) $$\begin{array}{c} v_{\varphi }=\left(\frac{\partial \Delta \varphi }{\partial \Phi }\right)d\Phi +\left(\frac{\partial \Delta \varphi }{\partial \Lambda }\right)d\Lambda +\left(\frac{\partial \Delta \varphi }{\partial \omega }\right)d\omega -\left(\Delta \varphi^{obs}-\;\Delta \varphi^{cal}\right)\\ v_{\lambda }=\left(\frac{\partial \Delta \lambda }{\partial \Phi }\right)d\Phi +\left(\frac{\partial \Delta \lambda }{\partial \Lambda }\right)d\Lambda +\left(\frac{\partial \Delta \lambda }{\partial \omega }\right)d\omega -\left(\Delta \lambda^{obs}-\Delta \lambda^{cal}\right) \end{array}$$

where *obs* and *cal* mean observed and calculated values, respectively.

The expressions given in Equation (3) are calculated based on the following relations, Equation (4) [14]:

(4) $$\begin{array}{c} \frac{\partial \Delta \varphi }{\partial \Phi }=-\omega \cdot \Delta t\cdot \sin\Phi \cdot \sin\left(\lambda -\Lambda \right)\\ \frac{\partial \Delta \varphi }{\partial \Lambda }=-\omega \cdot \Delta t\cdot \cos\Phi \cdot \cos\left(\lambda -\Lambda \right)\\ \frac{\partial \Delta \varphi }{\partial \omega }=\Delta t\cdot \cos\Phi \cdot \sin\left(\lambda -\Lambda \right)\\ \frac{\partial \Delta \lambda }{\partial \Phi }=\omega \cdot \Delta t\cdot \cos\Phi +\omega \cdot \Delta t\cdot \cos\left(\lambda -\Lambda \right)\cdot \tan\varphi \sin\Phi \\ \frac{\partial \Delta \lambda }{\partial \Lambda }=-\omega \cdot \Delta t\cdot \cos\Phi \cdot \sin\left(\lambda -\Lambda \right)\cdot \tan\varphi \\ \frac{\partial \Delta \lambda }{\partial \omega }=\Delta t\cdot (\sin\Phi -\cos\left(\lambda -\Lambda \right)\cdot \tan\varphi \cos\Phi ) \end{array}$$

For the purposes of this study, the sequential method was applied to determine the plate motion parameters. It involves several calculation steps. In the first step, the Φ, Λ, and *ω* parameters are determined based on the coordinates and velocities of two stations, here, the GNSS stations (station 1 + station 2) located on a given tectonic plate, adopted from the ITRF2014 [28] and available for users on the website http://itrf.ensg.ign.fr/ITRF_solutions/2014/ [44] (accessed on 8 June 2021).

**Figure 2:**
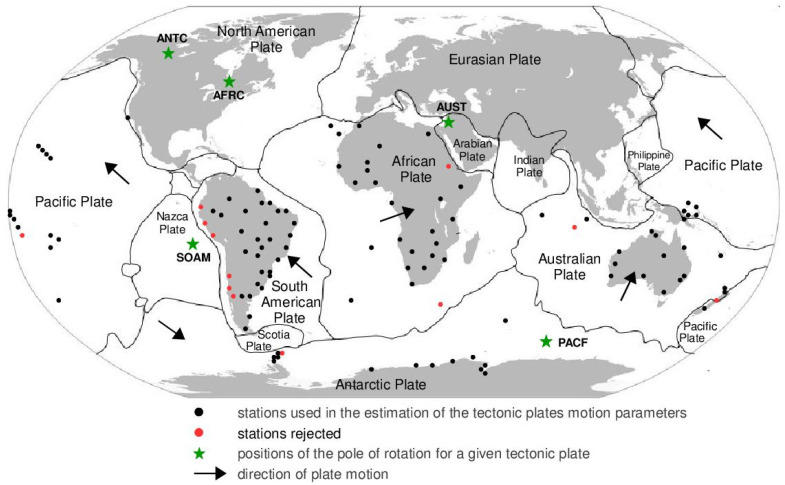
Distribution of the GNSS stations on tectonic plates and estimated positions of the pole of rotation for a given tectonic plate.

The next steps of the sequential method consist of adding further stations to the calculations, one by one, according to the scheme: station 1 + station 2, station 1 + station 2 + station 3, station 1 + station 2 + station 3 + ,…, station *n*, where *n* is the number of stations on the given plate as adopted in the solution. The number of stations added varies depending on the specific plate. In every subsequent step of the sequential method, the Φ, Λ, and *ω* parameters are calculated again. The application of the sequential method enables the obtainment of an increasingly stable adjustment, which is characterised by a decreasing formal error of the determined parameters. By increasing the number of stations, it arrives at a solution that, for a certain number of stations, is characterised by the high stability of the solution and minimum error values. A further increase in the number of stations results in the variability of the calculated motion parameter values within the limits of formal error, which is discussed in Section 3. The final values of the Φ, Λ, and *ω* parameters that were adopted and used in further analyses were those obtained from *n* stations (i.e., from the last step of the sequential method) for a given tectonic plate. The selection of the stations that are the basis for the determination of the motion parameters of specific tectonic plates should take into consideration the geophysical conditions of their location. Another factor that influences the accuracy of the solution is the geometrical configuration of the station network, which has been explained in [14,17]. Consistency with the previous determination of the Φ, Λ, and *ω* parameters for the SLR [37,38], DORIS [38,39], and VLBI [38,40] techniques were maintained, and the same manner of selecting stations was used. These assumptions were created based on the tests conducted during the realisation of previous studies. Thus, as in the previous studies, for example, [40,41], the stations should be located on a stable, uncracked area of the given plate, outside deformation zones. It is recommended that the stations should be distributed as evenly as possibly on the given tectonic plate. The concentration of a large number of stations in a small area does not significantly improve the accuracy and stability of the solution. The distance between the first two stations should equal approximately 60% of the distance between plate boundaries, and it should not be shorter than 50 km. The minimum timespan of observations conducted at a given station should be three years. This is required to meet the requirements of the rigid plate motion theory, which was discussed in [4]. The number of GNSS stations used in the calculations differs for specific plates: for the African plate, 25 stations were used; for the Australian plate, 20 stations were used; for the South American plate 29 stations were used; for the Antarctic plate, 13 stations were used; and finally, for the Pacific plate, 19 stations were used. As far as the Eurasian plate is concerned, which was analysed by Jagoda and Rutkowska [41], 120 GNSS stations were used (4 calculation scenarios were applied with 30 stations for each scenario). Figure 2 presents the stations that were used (black dots) and those that were rejected (red dots) from the solution and estimated positions (green stars) of the pole of rotation for particular plates (SOAM, South American; AFRC, African; PACF, Pacific; ANTC, Antarctic; AUST, Australian).

All calculations related to the determination of the Φ, Λ, and *ω* parameters were performed with use of the software in FORTRAN90, developed by the author, based on the least squares adjustment procedure. A block diagram of the adjustment of the plate motion parameters used in the author’s own software was shown in an earlier study [40] and is not repeated here. The adopted weights of observations were the formal errors in the determination of shifts of individual GNSS stations given in the ITRF2014 [28].

## 3. Results and Discussion

The positions and velocities of the GNSS stations in the ITRF2014 [28], determined by more than 21 years of GNSS observations that covered the period 1994.0–2015.1 [33], were the basis for the determination and analysis of the values of the Φ, Λ, and *ω* parameters that describe the movement of the five major tectonic plates: African, Australian, South American, Pacific, and Antarctic. A study by Jagoda and Rutkowska, on the largest tectonic plate, i.e., the Eurasian plate, has already been conducted and published in the literature [41]. In addition, the last of the major plates, the North American plate, should be analysed separately due to a very large amount of observational data. The North American plate is covered with a vast number of GNSS stations, which enables a detailed analysis of the area with very high tectonic activity, located along the boundary with the Pacific plate.

**Figure 3:**
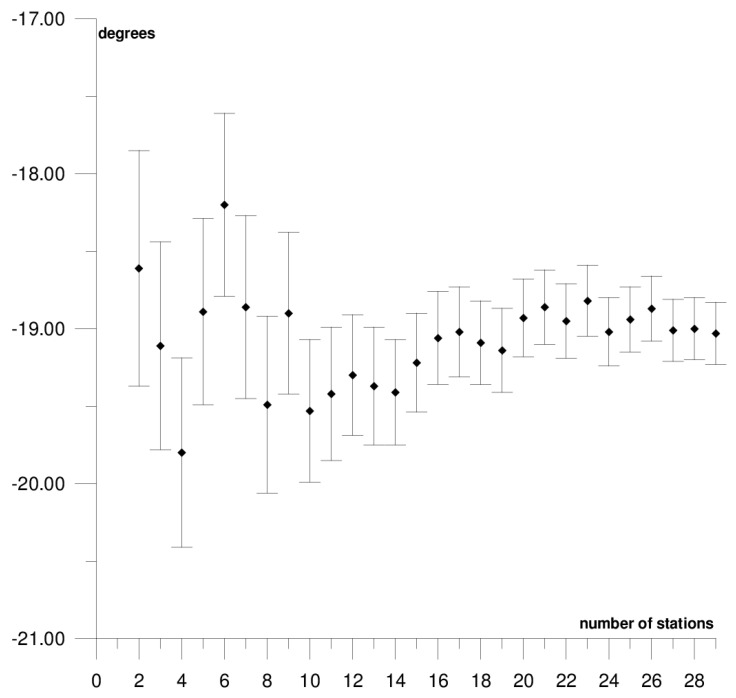
Results of sequential method for the Φ parameter for the South American plate.

**Figure 4:**
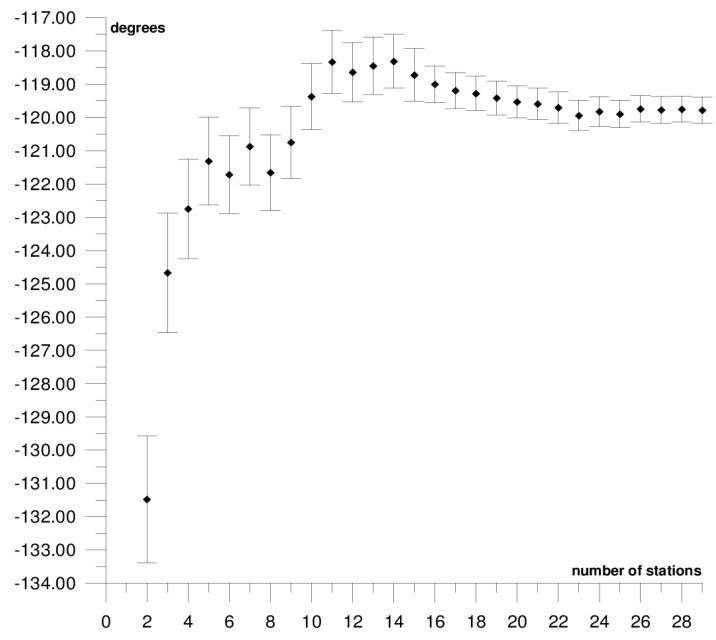
Results of the sequential method for the Λ parameter for the South American plate.

**Figure 5:**
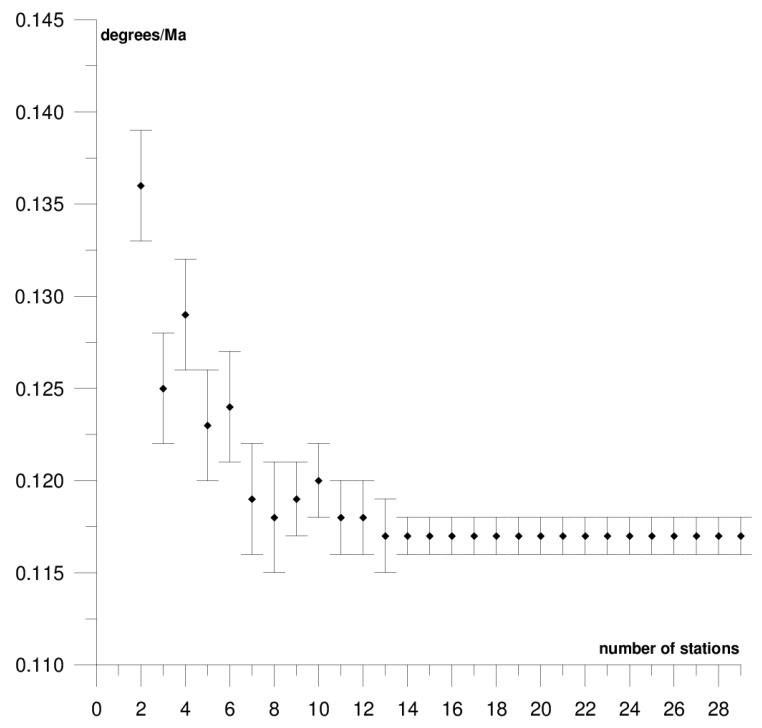
Results of the sequential method for the *ω* parameter for the South American plate.

**Figure 6:**
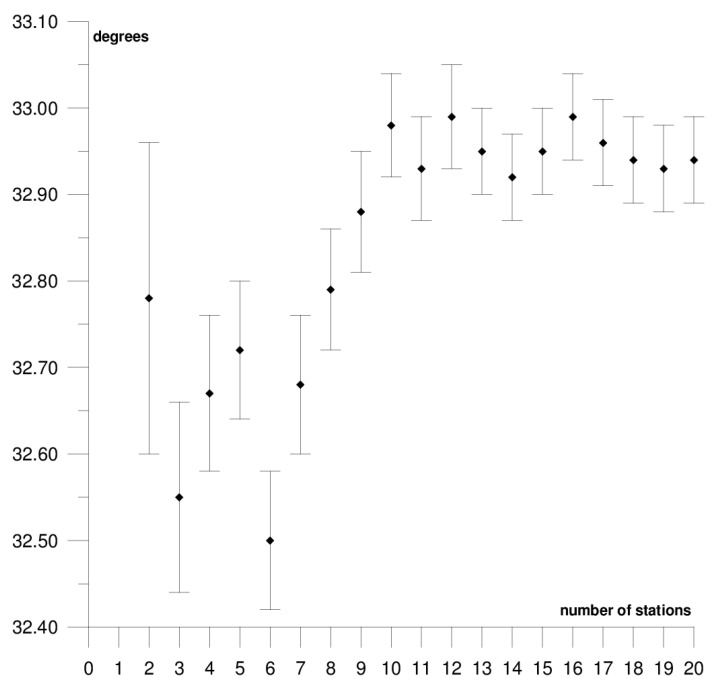
Results of the sequential method for the Φ parameter for the Australian plate.

**Figure 7:**
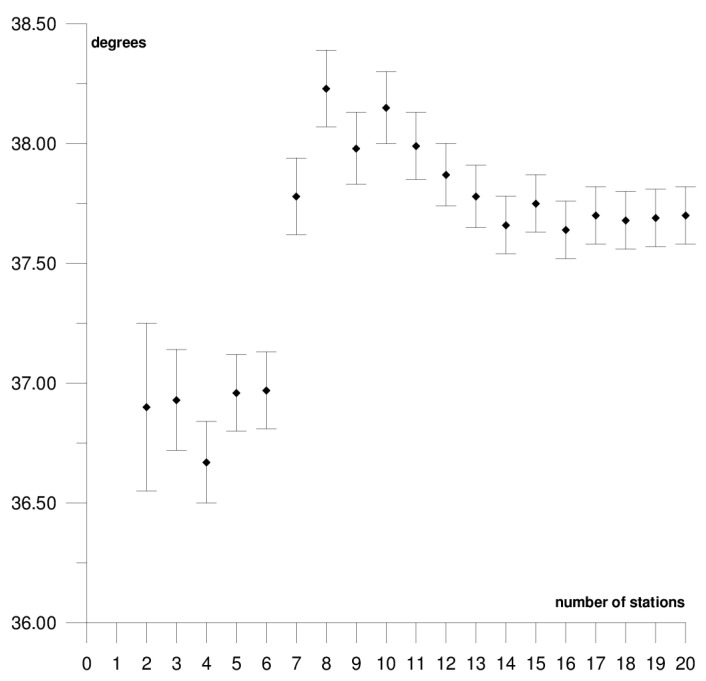
Results of the sequential method for the Λ parameter for the Australian plate.

**Figure 8:**
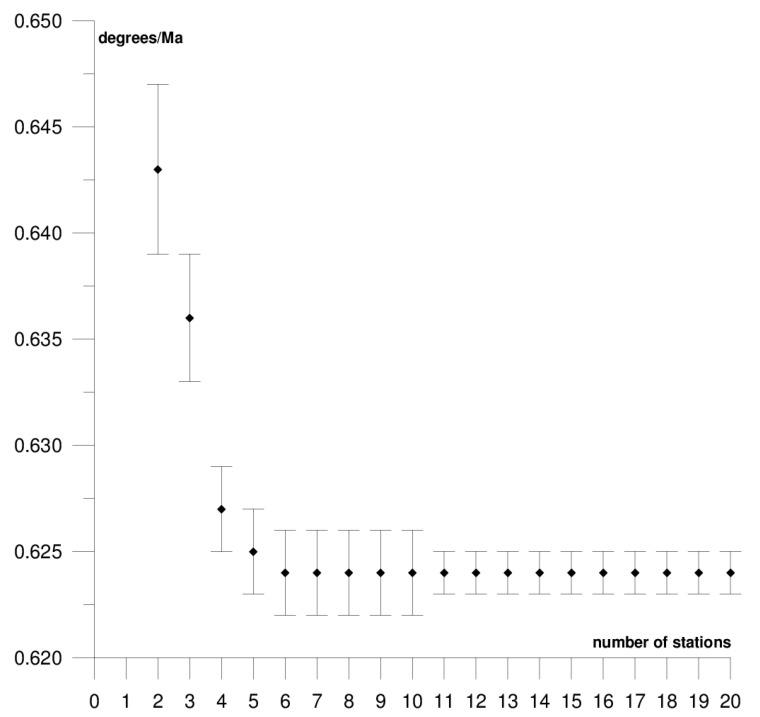
Results of the sequential method for the *ω* parameter for the Australian plate.

**Figure 9:**
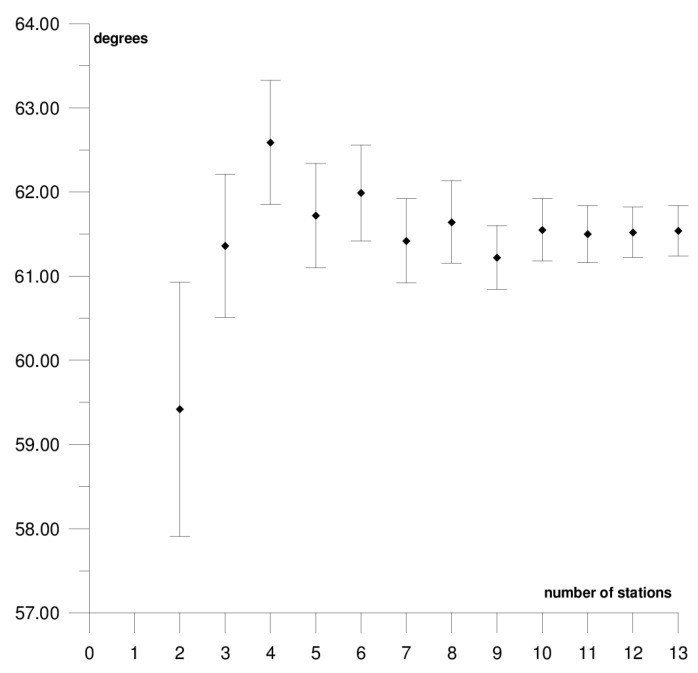
Results of the sequential method for the Φ parameter for the Antarctic plate.

**Figure 10:**
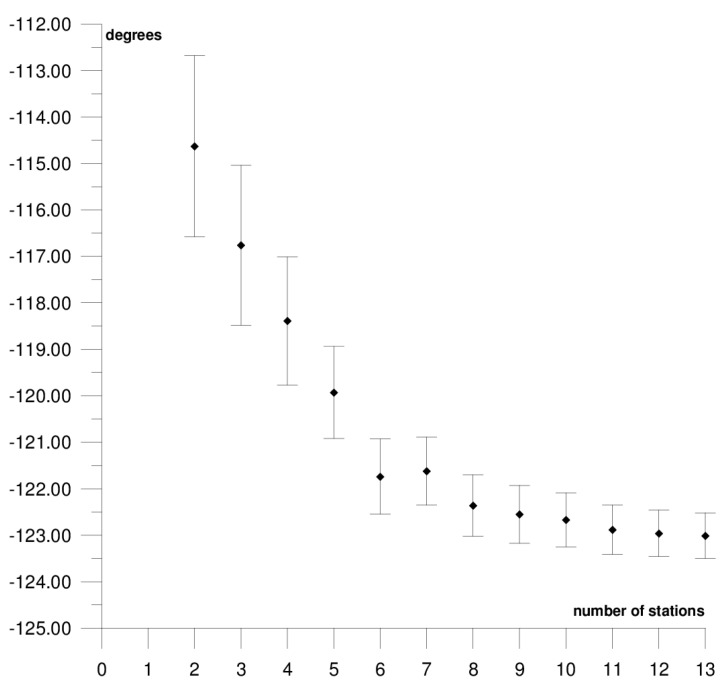
Results of the sequential method for the Λ parameter for the Antarctic plate.

**Figure 11:**
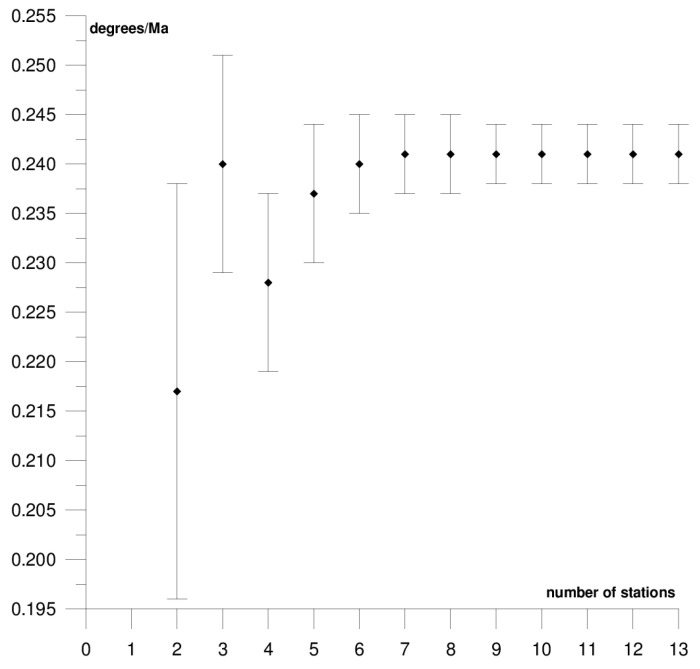
Results of the sequential method for the *ω* parameter for the Antarctic plate.

**Figure 12:**
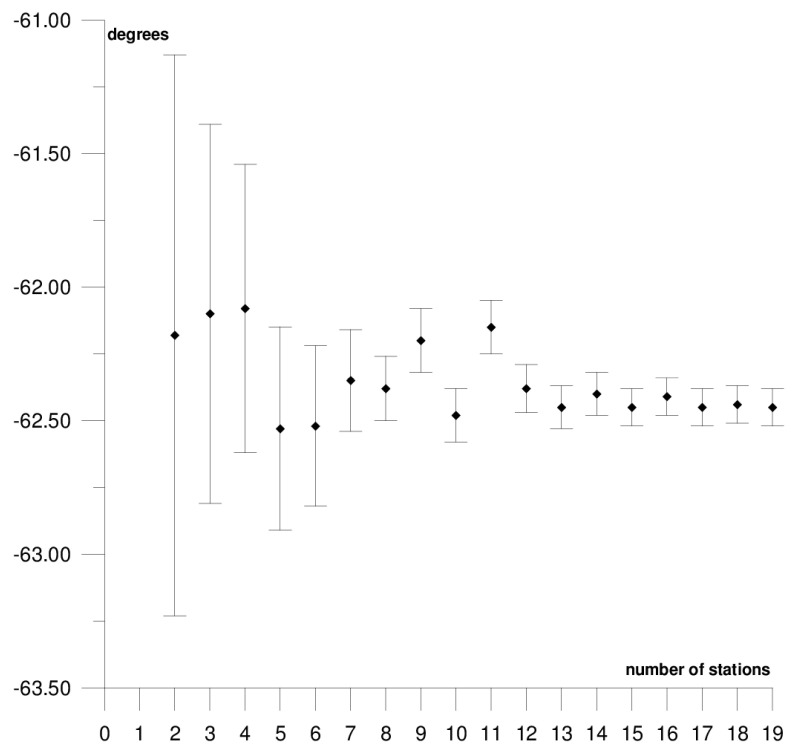
Results of the sequential method for the Φ parameter for the Pacific plate.

**Figure 13:**
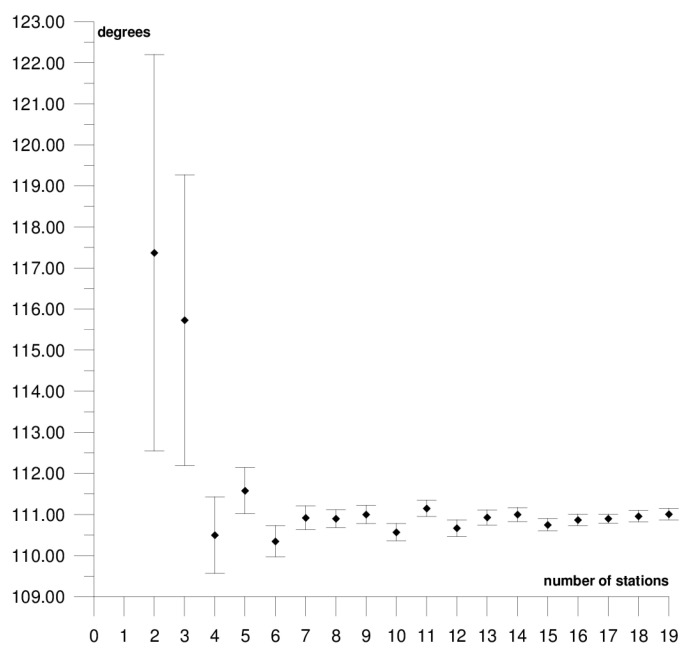
Results of the sequential method for the Λ parameter for the Pacific plate.

**Figure 14:**
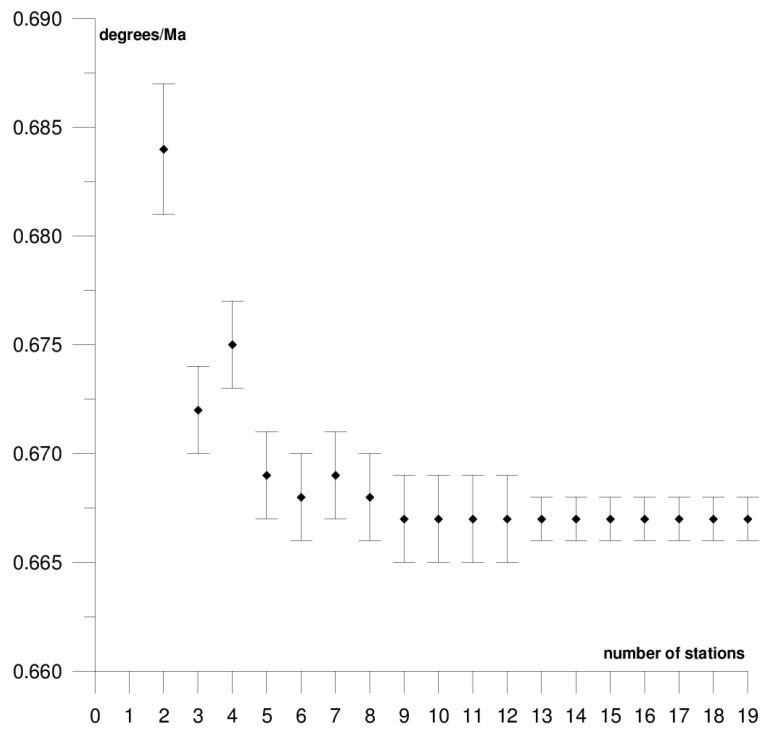
Results of the sequential method for the *ω* parameter for the Pacific plate.

**Figure 15:**
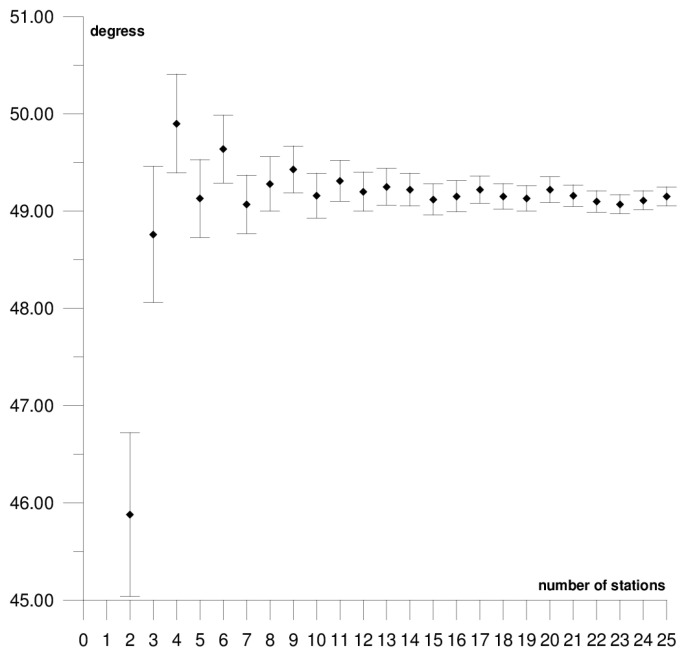
Results of the sequential method for the Φ parameter for the African plate.

**Figure 16:**
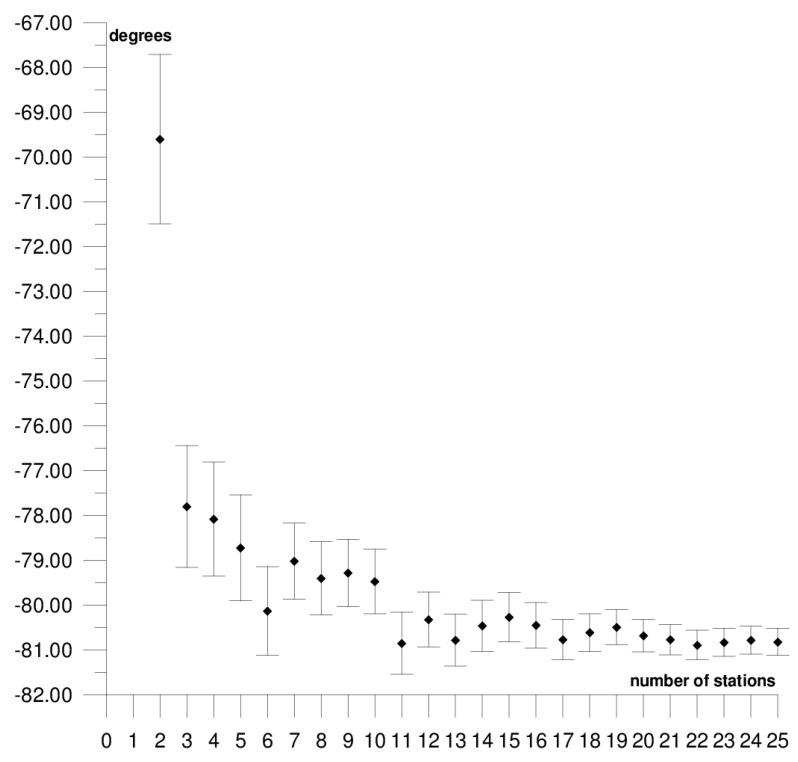
Results of the sequential method for the Λ parameter for the African plate.

**Figure 17:**
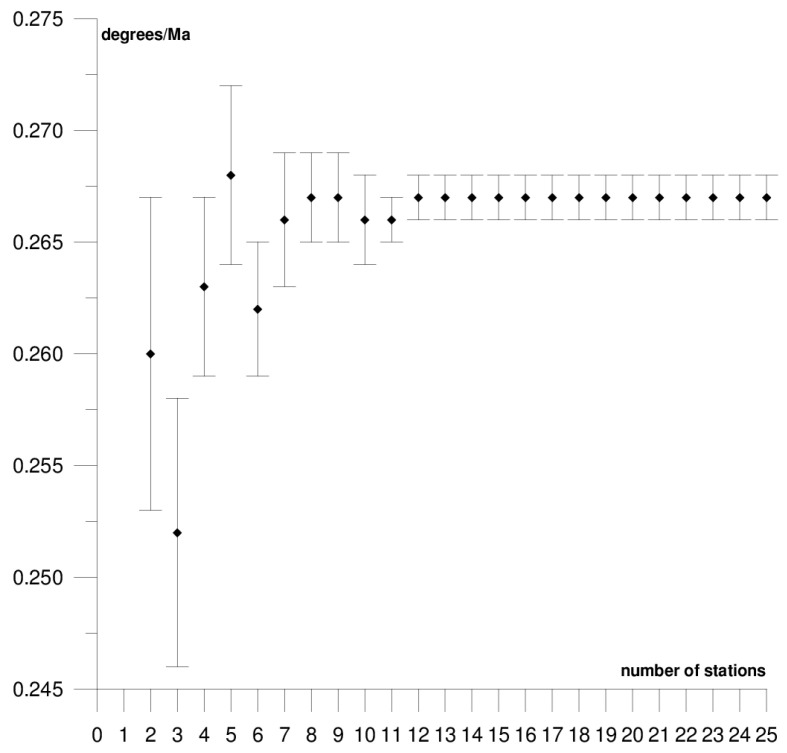
Results of the sequential method for the *ω* parameter for the African plate.

The results of each step of the sequential methods for the Φ, Λ, and *ω* parameter values are presented in Figure 3, Figure 4, Figure 5, Figure 6, Figure 7, Figure 8, Figure 9, Figure 10, Figure 11, Figure 12, Figure 13, Figure 14, Figure 15, Figure 16 and Figure 17 for each plate and parameter, both separately and in Appendix A, Appendix B, Appendix C, Appendix D and Appendix E, with the names of the stations used for the calculations. The final values of the Φ, Λ, and *ω* parameters and of formal errors adopted for further analyses correspond to the values from the last step of the sequential method.

The first plate that was analysed was the South American plate, which is bordered by the African and Nazca plates as well as several smaller ones with the oceanic lithosphere. It moves northwest at a rate of approximately 3 cm/year towards the North American plate, moving continuously away from Africa. It consists of three main geological units: the South American craton, which covers the northern, eastern, and central parts of the continent; the Palaeozoic Patagonian platform in the southeast; and the Andes, an alpine mountain range that stretches along the western boundary of the plate. In total, 29 GNSS stations were used to determine the motion parameters of the South American plate. The motion parameters calculated for all stations in total are: Φ = –19.03 ± 0.20°, Λ = −119.78 ± 0.39°, and *ω* = 0.117 ± 0.001°/Ma, which is presented, respectively, in Figure 3, Figure 4 and Figure 5 and in Appendix A. The comparison of the results with the values obtained in a previous study for the DORIS technique [39] demonstrated that the consistency of results was similar to the value of formal errors, and the differences were 1.27°, 1.57°, and 0.016°/Ma for the Φ, Λ, and *ω* parameters, respectively. For the other techniques, i.e., SLR and VLBI, the motion parameters of this plate were not calculated due to the lack of the required number of stations.

The solution becomes stable after 10, 11, and 7 steps of the sequential method, respectively, for the Φ, Λ, and *ω* parameters. At that stage, the changes in the values of the calculated parameters after adding subsequent stations to the process do not exceed the formal error. Hence, one may assume that the minimum number of stations required to determine the motion parameters of this plate is approximately 13. Due to high values of formal errors and discrepancies with the final values of the determined Φ, Λ, and *ω* parameters (please refer to Table 1), the following stations were rejected from the solutions: Valparaiso, Antuco, Concepcion, Quito III, Arequipa, and Callao (red dots on Figure 2). These stations accumulate interseismic strain associated with locking of the Peru-Chile megathrust, and therefore are moving east relative to the South American plate [45]. The Quito III station is located near the boundaries of four smaller plates: Cocos, Caribbean, Nazca, and North Andes. Including it in the solution causes a change in the Φ, Λ, and *ω* parameters, respectively, by approximately 3°, 10°, and 0.01°/Ma. The other stations: Callao, Arequipa, Valparaiso, Concepcion, and Antuco are situated along the boundary of the Nazca plate. Among them, the Callao station has the highest influence on the change in motion parameters, namely parameter Φ by approximately 6°, parameter Λ by approximately 11°, and parameter *ω* by −0.016°/Ma.

The motion parameters of the Australian plate were determined based on the position and velocity of 20 stations. Their final values are: Φ = 32.94 ± 0.05°, Λ = 37.70 ± 0.12°, and *ω* = 0.624 ± 0.001°/Ma. The results of the solution for each step of the sequential method are presented in Figure 6, Figure 7 and Figure 8 and in Appendix B. The solutions become stable for Φ, Λ, and *ω* parameters, respectively, after 10, 11, and 4 steps of the sequential method. Hence, one may assume that the minimum number of stations required to determine the motion parameters of this plate is approximately 12. The Australian plate is the most tectonically stable among all of the analysed plates, it moves northeast towards the Eurasian and Pacific plate at a rate of approximately 6–7 cm/year. The greater part of the Australian plate is occupied by the Precambrian Craton, called the Australian Craton, which is adjacent to the structure of the Flinders Ranges and the Barrier Ranges, and the structure of the Great Dividing Range [46]. The tectonic stability of this plate is reflected in the formal errors of the determined motion parameters, i.e., their values are lower than those of the remaining plates. Similar findings have been observed in previous studies on the SLR and DORIS techniques [37,38,39]. The comparison of the results with the values obtained in an earlier study on the SLR, DORIS, and VLBI techniques, the highest compatibility of results was found with the VLBI technique [40], and the lowest one with the SLR technique [37]. The differences in the determined Φ, Λ, and *ω* values for the GNSS and VLBI techniques are, respectively, 0.31°, 0.29°, and 0.007°/Ma, while, for the GNSS and SLR techniques, they are 1.52°, −1.78°, and 0.007°/Ma, respectively, for Φ, Λ, and *ω*. Two stations (Wellington and Coco Island) that disturb the result of calculating the Φ and Λ parameters by approximately 3° were found on the Australian plate (please refer to Table 1). Including these stations in the determination of the parameters also increases the formal errors of the parameters multiple times, therefore, these stations were rejected from the solutions. The Wellington station is located at the boundary with the Pacific plate near the so-called Alpine Fault, and the Coco Island station is located near the boundary with the Capricorn and Sunda small plates.

The next plate, the Antarctic plate is bordered by the African, South American, Australian, Pacific, Nazca, and Scotia plates. Within the plate, three main geological units can be distinguished: the Antarctic craton, which covers the eastern part of the continent; the Palaeozoic platform in the western part; and the Alpine fold zone of the Antarctic Peninsula. The Antarctic plate moves in the northeast direction (the part bordering the South American plate) and the southern direction (the part bordering the Australian plate) at a rate of about 1–1.5 cm/year. The following motion parameter values were obtained for the Antarctic plate: Φ = 61.54 ± 0.30°, Λ = −123.01 ± 0.49°, and *ω* = 0.241 ± 0.003°/Ma. The results of the sequential method are depicted in Figure 9, Figure 10 and Figure 11 and presented in Appendix C. The solution was based on 13 stations, whose positions are shown in Figure 2. The inclusion of the King George Island station located near the boundary with the Shetland microplate and the Scotia plate, in the area of the alpine fold of the Antarctic Peninsula, results in an approximately 10-fold increase in the formal errors and a change in the values of the Φ and Λ parameters by about 6° (Table 1); this station was not included in the calculations.

In general, the formal errors of the Φ, Λ, and *ω* parameters for the Antarctic plate were the highest among all of the analysed plates. The comparison of the obtained results to the solutions for the DORIS [39] and VLBI [40] techniques revealed that the compatibility was higher for DORIS. The differences in the determined values between the GNSS and DORIS techniques are 1.26° for Φ, 1.89° for Λ, and −0.009°/Ma for *ω*, while the differences between GNSS and VLBI are, respectively, 2.26°, 4.64°, and 0.025°/Ma. The stability of the solution of the Φ, Λ, and *ω* parameters appear after five steps of the sequential method. At that stage, the changes in the values of the calculated parameters, after the addition of another station to the process, do not exceed the formal error. Hence, one may assume that the minimum number of stations required to determine the motion parameters of this plate is approximately 7.

The sequential method for the motion parameters for the Pacific plate are presented in Figure 12, Figure 13 and Figure 14 and in Appendix D. It is the largest tectonic plate, and, within it, there are areas of very high tectonic activity, the so-called Ring of Fire. The Pacific plate moves northwest towards the Eurasian and Australian plates at a rate of approximately 6–10 cm/year. In the solution, 19 stations were included. Their locations are shown in Figure 2, and their names are listed in Appendix D. The final values of the motion parameters of the Pacific plate are: Φ = −62.45 ± 0.07°, Λ = 111.01 ± 0.14°, and *ω* = 0.667 ± 0.001°/Ma. Similar values were obtained in a previous study for the SLR, DORIS, and VLBI techniques [38]. The smallest differences were noted in the comparison with the SLR technique: 0.05° for the Φ parameter, 2.50° for the Λ parameter, and the value of the *ω* parameter was the same.

The stability of the solution of the Φ, Λ, and *ω* parameters appear, respectively, after 12, 15, and 5 steps of the sequential method. Adding further stations, up to 19 stations, resulted in a change of the Φ, Λ, and *ω* parameters by values that did not exceed formal errors. Hence, one may assume that the minimum number of stations required to determine the motion parameters of this plate is approximately 17. The solution took into consideration the Point Reyes Lig. station located on the North American continent that, on the one hand, its movement was compatible with that of the Pacific plate and it did not have a negative effect on the solution of the Φ, Λ, and *ω* parameters (please refer to Appendix D). On the other hand, the Nuku Alofa station was rejected, as it caused a multi-fold increase in the values of formal errors for the Φ, Λ, and *ω* parameters (please refer to Table 1) and changed their values by approximately 5° (Φ and Λ) and by 0.020°/Ma (*ω*).

Figure 15, Figure 16 and Figure 17 depict the results of the sequential method for the motion parameters of the African plate. It moves northeast towards the Eurasian and Arabian plates. The main geological unit is the Precambrian craton (the so–called African Megacraton) with a system of tectonic grabens, forming the East African Rift system. The sequential method was based on 25 GNSS stations, which are listed in Appendix E, and their locations are presented in Figure 2. The final values of the Φ, Λ, and *ω* parameters equal: Φ = 49.15 ± 0.10°, Λ = −80.82 ± 0.30°, and *ω* = 0.267 ± 0.001°/Ma. The obtained values of plate motion parameters in the first few steps of calculations were significantly divergent from the final results, as shown in Appendix E. Adding further stations in the calculation process led to the stabilisation of the results, until final values were reached based on all 25 stations. The stability of the results for the Φ, Λ, and *ω* parameters was noted, respectively, for 10, 7, and 7 stations used in the solution. Hence, the minimum number of stations required to ensure a stable solution is approximately 12 stations. The results were rather compatible with the values obtained in previous studies for the SLR [37] and DORIS [39] techniques, and the differences are, respectively, −1.63° and −0.38° for Φ, −4.00° and −1.70° for Λ, and −0.016°/Ma and 0.017°/Ma for *ω*. The Awra and Marion Island stations were rejected from the solution. The Awra station (located on the African continent near the boundary with the Arabian plate) contributed to a change in the Φ parameter by approximately 2.5°, Λ by approximately 2°, and *ω* by approximately 0.02°/Ma, while the Marion Island (located on an island near the boundary with the Antarctic plate) station changed the values by approximately 1.5° for Φ, by approximately 3.5° for Λ, and by approximately 0.01°/Ma for *ω* (please refer to Table 1). These stations also caused an approximately six-fold increase in the value of the formal errors in the determined parameters. The following stations are included in the solution: Addis Ababa, Mbarara, Tanzania CGPS, and Richardsbay. Although they are located on the Somalia and Lwandle small plates, they do not have a negative effect on the results of the solution (Appendix E).

### Comparison with Geological Model NNR-MORVEL56 and Geodetic ITRF2014 Plate Motion Model (PMM)

Geological models are developed based on geophysical observations, such as sea floor spreading rates, earthquake slip vectors, and transform fault azimuths. The most commonly used geological model is NNR-NUVEL1A [24] that originates from NNR-NUVEL1 [23]. It has recently been replaced with a new model, namely NNR-MORVEL56 [26], which is considered to be better than NNR-NUVEL1A. The NNR-MORVEL56 model was determined from more and higher quality spreading rates and azimuths. Moreover, it excluded circum-Pacific data (earthquake slip vectors and Pacific North America spreading rates) that were biased measures of relative plate velocity, addressed in [26]. The comparison of the results of the solutions of motion parameters of the specific tectonic plates with the NNR-MORVEL56 geological model should be approached with caution. The observations that are used for creating geological models are limited to plate boundaries, where local deformations occur quite often, and therefore the obtained results are not always representative of the movement of the whole plate. Moreover, geological models provide an average movement of individual plates across a very long period of time, which may range from hundreds of thousands to millions of years, and due to this, they may not reflect the potential current speeding up or slowing down of specific tectonic plates, which, however, are recorded by very precise space techniques: SLR, DORIS, VLBI, and GNSS. Nevertheless, the ITRF2000 was defined based on the tectonic plate movement obtained from the NNR-NUVEL1A geological model. Subsequent solutions of the ITRF were adopted to the previous ones: ITRF2005 to ITRF2000, ITRF2008 to ITRF2005, and ITRF2014 to ITRF2008, but they are still indirectly linked to NNR-NUVEL1A. The question about the use of the new geological model, i.e., the NNR-MORVEL56, in future solutions of the ITRF, is still important and was discussed in [28]. To date, it has been found that the angular velocities in NNR-MORVEL56 differ significantly from those in NNR-NUVEL1A for all plates [5,26]. A detailed comparative analysis of these models was conducted by Argus et al. [26] and it will not be discussed here. However, the comparison of the motion parameters of individual plates that were determined in this study with the NNR-MORVEL56 model is presented in Table 2.

The calculated differences between the values of the Φ, Λ, and *ω* parameters for the NNR-MORVEL 56 model and the results of the solution presented in this study revealed that the most similar values were obtained for the Australian plate: the Φ parameter differed by approximately 1°, the Λ parameter by approximately 0.2°, and the *ω* parameter by 0.008°/Ma, which corresponded to approximately 1 mm/year. A difference of approximately 1° for the Φ parameter was also obtained for the Pacific plate, although for the Λ parameter, the difference was higher and approximately 4°, while the angular rotation speed, *ω*, differed by approximately −0.02°/Ma, which corresponded to approximately 2 mm/year. Regarding the South American and Antarctic plates, the difference in the values of the Φ parameter was approximately 4°, and for the Λ parameter, approximately 7° (the South American plate) and approximately 5° (the Antarctic plate). The angular rotation speed *ω* differed by −0.008°/Ma for the South American plate and by 0.009°/Ma for the Antarctic plate, which corresponded to approximately 1 mm/year. The NNR-MORVEL56 model does not present the results for the African plate, which distinguishes two plates within it, namely Nubia and Somalia; hence, the inability to compare the obtained results. Nubia covers approximately 95% of the surface area of the African continent and the area to the West, towards the boundary of the South American plate, while the Somalia plate covers about 5% of the continent (the Eastern part) and the area to the East to the boundary with the Australian plate and the smaller tectonic plate, i.e., the Indian plate.

The ITRF2014 PMM [5] is the geodetic model describing the motion of 11 tectonic plates (the major plates and several selected small plates). It is dedicated to the currently in force ITRF2014 frame [28]. In developing it, horizontal velocities of a subset of the ITRF2014 stations of all space techniques in a combined solution (SLR + DORIS + VLBI + GNSS) were used, localized away from plate boundaries and deforming zones. For the South American plate, it was a total of 30 stations apart from the GNSS stations also 2 DORIS ones); for the Australian plate, it was 36 stations (including five DORIS, five VLBI, and three SLR stations); for the Pacific plate, it was 18 stations (including four DORIS, three VLBI, and one SLR); for the Antarctic plate, it was seven stations (GNSS only). Similar to the NNR-MORVEL 56 model, no parameters were determined for the African plate. In the ITRF2014 PMM, the plate motion was described providing specific elements of the pole of rotation: *ω*_x_, *ω*_y_, *ω*_z_, and the angular rotation speed *ω*. In order to compare the results, the values of geographical latitude (Φ) and longitude (Λ) of the pole of rotation determined in this study for each plate were calculated with the use of the formulas (Equation (1)) into *ω*_x_, *ω*_y_, and *ω*_z_. The comparison is presented in Table 3. As it can be seen from that table, the largest differences are noted for the South American plate, whereas small differences can be noted for the Australian plate. From the analysis of separate components of the pole of rotation (*ω*_x_, *ω*_y_, and *ω*_z_), it becomes evident that the largest differences for the *ω*_x_ component occur for the South American plate (−0.082 mas/year) and the Antarctic plate (−0.023 mas/year); for the *ω*_y_ component, the largest differences occur for the South American plate (0.050 mas/year) and the Australian plate (0.029 mas/year); while for the *ω*_z_ component, the largest differences occur for the Antarctic plate (−0.088 mas/year) and the Pacific plate (−0.057 mas/year). For the rotation angular velocity (*ω*), the biggest difference is in the case of the Antarctic plate (−0.022°/Ma) and the Pacific plate (0.012°/Ma), which corresponds to approximately 2 mm/year and 1 mm/year, respectively. For the remaining plates the differences do not exceed 1 mm/year.

In general, it can be stated that the agreement with the NNR-MORVEL56 and ITRF2014 PMM models is good for all plates.

## 4. Conclusions

In this study, the motion parameters, i.e., the latitude (Φ) and longitude (Λ) of the rotation pole, and the angular rotation speed (*ω*) of five major lithospheric plates (South American, African, Australian, Antarctic, and Pacific plates) were determined. The observational material includes the positions and velocities of 106 highly quality GNSS stations in the ITRF2014. As many as 29 of these stations are located on the South American plate, 13 stations on the Antarctic plate, 20 stations on the Australian plate, 19 stations on the Pacific plate, and 25 stations on the African plate. The most accurate solutions of the Φ, Λ, and *ω* parameters were determined for the Australian and Pacific plates, while the least accurate were determined for the Antarctic plate. The comparison of the obtained results to previously conducted research on the SLR [37,38], DORIS [38,39], and VLBI [38,40] techniques revealed high compatibility. The most similar results were obtained for the comparison with the VLBI technique for the Australian plate, the SLR technique for the Pacific plate, and the DORIS technique for the African, Antarctic, and South American plates. The most accurate solutions for the Φ, Λ, and *ω* parameters were obtained with the GNSS technique as compared with the other techniques. Because of the dense coverage of the Earth with GNSS stations, this is the only space technique that offers the possibility to determine the motion parameters of all the major lithospheric plates. The applied sequential method allowed us to define the minimum number of stations that ensured a stable solution and to indicate the stations that negatively affected the result of the solution. The minimum number of stations that should be used to determine the Φ, Λ, and *ω* parameters to guarantee a stable solution differs depending on the individual plates. For the South American and Australian plates, this number is approximately 13 stations; 7 stations for the Antarctic plate; 17 stations for the Pacific plate; and 12 stations for the African plate. Including a larger number of stations in the calculations does not have a significant influence on the values of the determined Φ, Λ, and *ω* parameters and formal errors. A total number of 12 stations that disturbed the solutions of the Φ, Λ, and *ω* parameters and increased the values of formal errors were found; these stations were rejected from the solution. Six of these stations are located on the South American plate (Valparaiso, Antuco, Concepcion, Quito III, Arequipa, and Callao), two stations on the Australian (Wellington and Coco Island) and African (Awra and Marion Island) plates, and one on the Antarctic (King George Island) and Pacific (Nuku Alofa) plates. Including them in the solution leads to a change in the Φ and Λ parameters in the range of 2–11°, and of the *ω* parameter in the range from −0.016 to 0.058°/Ma.

The values that were the most similar to the current geological model NNR-MORVEL56 [26] were obtained for the Australian plate (the differences do not exceed 1° for the Φ and the Λ parameters, and 0.008°/Ma for the *ω* parameter). The differences for the other plates are higher, ranging from approximately 1 to 7° for the Φ and the Λ parameters, and from −0.016 to 0.008°/Ma for the *ω* parameter. These differences may indicate a current slowing down or speeding up of the movement of certain tectonic plates, which are detected by the very precise GNSS space technique. Comparing the results with the geodetic model ITRF2014 PMM [5], one can find a high agreement between them, the largest differences are found for the South American plate (*ω*_x_ and *ω*_y_ components) and the Antarctic plate (*ω*_z_ component). The rotation angular velocity (*ω*) differs within the range from −0.022°/Ma (the Antarctic plate) to 0.012°/Ma (the Pacific plate).

## Figures and Tables

**Table 1:** Stations rejected from the solutions.

Plate Name	Name of the Station	Φ (°)	Λ (°)	*ω* (°/Ma)
South American	Valparaiso	−14.47 ± 2.41	−117.08 ± 5.24	0.131 ± 0.011
	Antuco	−18.05 ± 1.06	−117.53 ± 2.54	0.110 ± 0.010
	Concepcion	−15.34 ± 2.39	−115.15 ± 5.43	0.132 ± 0.014
	Quito III	−16.09 ± 0.82	−129.78 ± 1.91	0.118 ± 0.014
	Arequipa	−16.06 ± 1.07	−127.45 ± 2.51	0.124 ± 0.008
	Callao	−13.01 ± 1.46	−130.48 ± 3.35	0.133 ± 0.012
Australian	Wellington	30.23 ± 1.03	34.14 ± 1.51	0.633 ± 0.005
	Coco Island	31.00 ± 1.27	41.08 ± 2.56	0.583 ± 0.006
Pacific	Nuku Alofa	−56.97 ± 2.15	116.27 ± 1.97	0.647 ± 0.010
Antarctic	King George Island	56.56 ± 3.40	−128.76 ± 3.07	0.183 ± 0.022
African	Awra	46.59 ± 0.47	−78.73 ± 2.02	0.250 ± 0.006
	Marion Island	50.64 ± 0.51	−84.34 ± 1.23	0.254 ± 0.005

**Table 2:** Comparison with the NNR-MORVEL56 model.

Plate Name	NNR-MORVEL56 (1) Φ (°), Λ (°), *ω* (°/Ma)	This Paper (2) Φ (°), Λ (°), *ω* (°/Ma)	Differences (1)–(2) Φ (°), Λ (°), *ω* (°/Ma)
South American	−22.62	−19.03 ± 0.20	−3.59
	−112.83	−119.78 ± 0.39	6.95
	0.109	0.117 ± 0.001	−0.008
Australian	33.86	32.94 ± 0.05	0.92
	37.94	37.70 ± 0.12	0.24
	0.632	0.624 ± 0.001	0.008
Pacific	−63.58	−62.45 ± 0.07	−1.13
	114.70	111.01 ± 0.14	3.69
	0.651	0.667 ± 0.001	−0.016
Antarctic	65.42	61.54 ± 0.30	3.88
	−118.11	−123.01 ± 0.49	4.90
	0.250	0.241 ± 0.003	0.009

**Table 3:** Comparison with the ITRF2014 PMM.

Plate Name	ITRF2014 PMM (1) *ω*_x_, *ω*_y_, *ω*_z_ (mas/yr), *ω* (°/Ma)	This Paper (2) *ω*_x_, *ω*_y_, *ω*_z_ (mas/yr), *ω* (°/Ma)	Differences (1)–(2) *ω*_x_, *ω*_y_, *ω*_z_ (mas/yr), *ω* (°/Ma)
South American	−0.270 ± 0.006	−0.188 ± 0.003	−0.082
	−0.301 ± 0.006	−0.351 ± 0.003	0.050
	−0.140 ± 0.003	−0.136 ± 0.002	−0.004
	0.119 ± 0.001	0.117 ± 0.001	0.002
Australian	1.510 ± 0.004	1.492 ± 0.004	0.018
	1.182 ± 0.004	1.153 ± 0.004	0.029
	1.215 ± 0.004	1.222 ± 0.004	−0.007
	0.631 ± 0.001	0.624 ± 0.001	0.007
Pacific	−0.409 ± 0.003	−0.410 ± 0.004	0.001
	1.047 ± 0.004	1.066 ± 0.004	−0.019
	−2.169 ± 0.004	−2.112 ± 0.004	−0.057
	0.679 ± 0.001	0.667 ± 0.001	0.012
Antarctic	−0.248 ± 0.004	−0.225 ± 0.002	−0.023
	−0.324 ± 0.004	−0.347 ± 0.003	0.023
	0.675 ± 0.008	0.763 ± 0.021	−0.088
	0.219 ± 0.002	0.241 ± 0.003	−0.022

## Data Availability

Not applicable.

## Appendix A

Determined plate motion parameters and their formal errors of the South American plate for the GNSS network using the sequential method.

**Table:**

No.	Name of the Station	Φ(°)	Λ(°)	ω(°/Ma)
2	Brasilia + Forteleza	−18.61 ± 0.76	−131.48 ± 1.91	0.136 ± 0.003
3	2 + Buenos Aires	−19.11 ± 0.67	−124.67 ± 1.80	0.125 ± 0.003
4	3 + Rio Grande	−19.80 ± 0.61	−122.75 ± 1.49	0.129 ± 0.003
5	4 + La Plata	−18.89 ± 0.60	−121.31 ± 1.32	0.123 ± 0.003
6	5 + Bahia Blanca	−18.20 ± 0.59	−121.72 ± 1.18	0.124 ± 0.003
7	6 + Lihue Calel	−18.86 ± 0.59	−120.87 ± 1.16	0.119 ± 0.003
8	7 + Curitiba-Para	−19.49 ± 0.57	−121.66 ± 1.14	0.118 ± 0.003
9	8 + Presidente Prud	−18.90 ± 0.52	−120.75 ± 1.09	0.119 ± 0.002
10	9 + Manaus	−19.53 ± 0.46	−119.37 ± 0.99	0.120 ± 0.002
11	10 + Porto Alegre	−19.42 ± 0.43	−118.33 ± 0.95	0.118 ± 0.002
12	11 + Recif	−19.30 ± 0.39	−118.64 ± 0.89	0.118 ± 0.002
13	12 + Cananeia	−19.37 ± 0.38	−118.45 ± 0.86	0.117 ± 0.002
14	13 + Belem	−19.41 ± 0.34	−118.31 ± 0.81	0.117 ± 0.001
15	14 + Porto Velho	−19.22 ± 0.32	−118.72 ± 0.79	0.117 ± 0.001
16	15 + Macapa	−19.06 ± 0.30	−119.00 ± 0.55	0.117 ± 0.001
17	16 + Ilha Solteira	−19.02 ± 0.29	−119.19 ± 0.54	0.117 ± 0.001
18	17 + Boa Vista	−19.09 ± 0.27	−119.28 ± 0.52	0.117 ± 0.001
19	18 + Imbituba	−19.14 ± 0.27	−119.41 ± 0.51	0.117 ± 0.001
20	19 + Sao Gabriel	−18.93 ± 0.25	−119.53 ± 0.48	0.117 ± 0.001
21	20 + Sao Luis	−18.86 ± 0.24	−119.59 ± 0.47	0.117 ± 0.001
22	21 + Salvador Capita	−18.95 ± 0.24	−119.70 ± 0.47	0.117 ± 0.001
23	22 + Rio Branco	−18.82 ± 0.23	−119.94 ± 0.45	0.117 ± 0.001
24	23 + Palmas	−19.02 ± 0.22	−119.82 ± 0.44	0.117 ± 0.001
25	24 + Kourou	−18.94 ± 0.21	−119.90 ± 0.41	0.117 ± 0.001
26	25 + Yacuiba	−18.87 ± 0.21	−119.74 ± 0.40	0.117 ± 0.001
27	26 + Cuiba	−19.01 ± 0.20	−119.77 ± 0.40	0.117 ± 0.001
28	27 + Punta Arenas	−19.00 ± 0.20	−119.75 ± 0.39	0.117 ± 0.001
29	28 + Iquitos	**−19.03 ± 0.20**	**−119.78 ± 0.39**	**0.117 ± 0.001**

## Appendix B

Determined plate motion parameters and their formal errors of the Australian plate for the GNSS network using the sequential method.

**Table:**

No.	Name of the Station	Φ(°)	Λ(°)	ω(°/Ma)
2	Melbourne Obser + Yarragadee2	32.78 ± 0.18	36.90 ± 0.35	0.643 ± 0.004
3	2 + Christmas Islan	32.55 ± 0.11	36.93 ± 0.21	0.636 ± 0.003
4	3 + Warkworth	32.67 ± 0.09	36.67 ± 0.17	0.627 ± 0.002
5	4 + Darwin I	32.72 ± 0.08	36.96 ± 0.16	0.625 ± 0.002
6	5 + Alice Springs	32.50 ± 0.08	36.97 ± 0.16	0.624 ± 0.002
7	6 + Diego Garcia	32.68 ± 0.08	37.78 ± 0.16	0.624 ± 0.002
8	7 + Noumea	32.79 ± 0.07	38.23 ± 0.16	0.624 ± 0.002
9	8 + Karratha	32.88 ± 0.07	37.98 ± 0.15	0.624 ± 0.002
10	9 + Lae–Universit	32.98 ± 0.06	38.15 ± 0.15	0.624 ± 0.002
11	10 + Tidbinbilla1	32.93 ± 0.06	37.99 ± 0.14	0.624 ± 0.001
12	11 + Koumac	32.99 ± 0.06	37.87 ± 0.13	0.624 ± 0.001
13	12 + Sydney	32.95 ± 0.05	37.78 ± 0.13	0.624 ± 0.001
14	13 + Katherine-Nor1	32.92 ± 0.05	37.66 ± 0.12	0.624 ± 0.001
15	14 + Townsville-Ca	32.95 ± 0.05	37.75 ± 0.12	0.624 ± 0.001
16	15 + Auckland	32.99 ± 0.05	37.64 ± 0.12	0.624 ± 0.001
17	16 + Ceduna	32.96 ± 0.05	37.70 ± 0.12	0.624 ± 0.001
18	17 + Parkes	32.94 ± 0.05	37.68 ± 0.12	0.624 ± 0.001
19	18 + Perth	32.93 ± 0.05	37.69 ± 0.12	0.624 ± 0.001
20	19 + Mount Stromlo	**32.94 ± 0.05**	**37.70 ± 0.12**	**0.624 ± 0.001**

## Appendix C

Determined plate motion parameters and their formal errors of the Antarctic plate for the GNSS network using the sequential method.

**Table:**

No.	Name of the Station	Φ(°)	Λ(°)	ω(°/Ma)
2	Syowa + Rothera	59.42 ± 1.51	−114.63 ± 1.95	0.217 ± 0.021
3	2 + O’Higgins	61.36 ± 0.85	−116.76 ± 1.72	0.240 ± 0.011
4	3 + Casey	62.59 ± 0.74	−118.39 ± 1.38	0.228 ± 0.009
5	4 + Vernadski	61.72 ± 0.62	−119.93 ± 0.99	0.237 ± 0.007
6	5 + Mount Fleming	61.99 ± 0.57	−121.74 ± 0.81	0.240 ± 0.005
7	6 + Ile des Petrels	61.42 ± 0.50	−121.62 ± 0.73	0.241 ± 0.004
8	7 + Mac Murdo	61.64 ± 0.49	−122.36 ± 0.66	0.241 ± 0.004
9	8 + Mawson	61.22 ± 0.38	−122.55 ± 0.62	0.241 ± 0.003
10	9 + Palmer	61.55 ± 0.37	−122.67 ± 0.58	0.241 ± 0.003
11	10 + Sanae	61.50 ± 0.34	−122.88 ± 0.53	0.241 ± 0.003
12	11 + Fishtail Point	61.52 ± 0.30	−122.96 ± 0.50	0.241 ± 0.003
13	12 + Cape Roberts	**61.54 ± 0.30**	**−123.01 ± 0.49**	**0.241 ± 0.003**

## Appendix D

Determined plate motion parameters and their formal errors of the African plate for the GNSS network using the sequential method.

**Table:**

No.	Name of the Station	Φ(°)	Λ(°)	ω(°/Ma)
2	Kauai + Papeete (Tahiti)	−62.18 ± 1.05	117.37 ± 4.83	0.684 ± 0.003
3	2 + Maui I	−62.10 ± 0.71	115.73 ± 3.54	0.672 ± 0.002
4	3 + Dunedin	−62.08 ± 0.54	110.50 ± 0.93	0.675 ± 0.002
5	4 + Mauna Kea	−62.53 ± 0.38	111.58 ± 0.56	0.669 ± 0.002
6	5 + Rikitea (Ile Man)	−62.52 ± 0.30	110.35 ± 0.38	0.668 ± 0.002
7	6 + Niue Island	−62.35 ± 0.19	110.92 ± 0.29	0.669 ± 0.002
8	7 + American Samoa	−62.38 ± 0.12	110.90 ± 0.22	0.668 ± 0.002
9	8 + Apia	−62.20 ± 0.12	111.00 ± 0.22	0.667 ± 0.002
10	9 + Tubuai	−62.48 ± 0.10	110.57 ± 0.21	0.667 ± 0.002
11	10 + Futuna	−62.15 ± 0.10	111.15 ± 0.20	0.667 ± 0.002
12	11 + Point Reyes Lig	−62.38 ± 0.09	110.67 ± 0.20	0.667 ± 0.002
13	12 + Chatham Island	−62.45 ± 0.08	110.93 ± 0.18	0.667 ± 0.001
14	13 + Betio Island	−62.40 ± 0.08	111.00 ± 0.17	0.667 ± 0.001
15	14 + Kwajalein Atoll	−62.45 ± 0.07	110.75 ± 0.15	0.667 ± 0.001
16	15 + Nauru	−62.41 ± 0.07	110.87 ± 0.14	0.667 ± 0.001
17	16 + Majuro	−62.45 ± 0.07	110.90 ± 0.11	0.667 ± 0.001
18	17 + Pohnpei	−62.44 ± 0.07	110.96 ± 0.14	0.667 ± 0.001
19	18 + Honolulu	**−62.45 ± 0.07**	**111.01 ± 0.14**	**0.667 ± 0.001**

## Appendix E

Determined plate motion parameters and their formal errors of the African plate for the GNSS network using the sequential method.

**Table:**

No.	Name of the Station	Φ(°)	Λ(°)	ω(°/Ma)
2	Cotonou + Lusaka	45.88 ± 0.84	−69.60 ± 1.89	0.260 ± 0.007
3	2 + Addis Ababa Uni	48.76 ± 0.70	−77.80 ± 1.36	0.252 ± 0.006
4	3 + Dakar Universit	49.90 ± 0.51	−78.08 ± 1.27	0.263 ± 0.004
5	4 + Sutherland	49.13 ± 0.40	−78.72 ± 1.18	0.268 ± 0.004
6	5 + Gough Island	49.64 ± 0.35	−80.13 ± 0.99	0.262 ± 0.003
7	6 + Gao	49.07 ± 0.30	−79.02 ± 0.85	0.266 ± 0.003
8	7 + Tamale	49.28 ± 0.28	−79.40 ± 0.82	0.267 ± 0.002
9	8 + Yamoussoukro	49.43 ± 0.24	−79.28 ± 0.75	0.267 ± 0.002
10	9 + Springbok	49.16 ± 0.23	−79.47 ± 0.72	0.266 ± 0.002
11	10 + Richardsbay	49.31 ± 0.21	−80.85 ± 0.69	0.266 ± 0.001
12	11 + Walvis Ba	49.20 ± 0.20	−80.32 ± 0.61	0.267 ± 0.001
13	12 + Helwan	49.25 ± 0.19	−80.78 ± 0.58	0.267 ± 0.001
14	13 + Hartebeesthoek	49.22 ± 0.17	−80.46 ± 0.57	0.267 ± 0.001
15	14 + Telecomm Centre	49.12 ± 0.16	−80.27 ± 0.55	0.267 ± 0.001
16	15 + Hermanus	49.15 ± 0.16	−80.45 ± 0.51	0.267 ± 0.001
17	16 + De Aar	49.22 ± 0.14	−80.77 ± 0.45	0.267 ± 0.001
18	17 + Ouagadougou	49.15 ± 0.13	−80.61 ± 0.42	0.267 ± 0.001
19	18 + Maspalomas	49.13 ± 0.13	−80.49 ± 0.39	0.267 ± 0.001
20	19 + Santa Cruz	49.22 ± 0.13	−80.68 ± 0.36	0.267 ± 0.001
21	20 + Saint Helena	49.16 ± 0.11	−80.77 ± 0.34	0.267 ± 0.001
22	21 + Tanzania CGPS	49.10 ± 0.11	−80.89 ± 0.33	0.267 ± 0.001
23	22 + Simonstown	49.07 ± 0.10	−80.83 ± 0.31	0.267 ± 0.001
24	23 + Mbarara	49.11 ± 0.10	−80.78 ± 0.31	0.267 ± 0.001
25	24 + Rabat	**49.15 ± 0.10**	**−80.82 ± 0.30**	**0.267 ± 0.001**
